# Phylogenomics of *Tetraopes* longhorn beetles unravels their evolutionary history and biogeographic origins

**DOI:** 10.1038/s41598-024-57827-z

**Published:** 2024-03-27

**Authors:** Nayeli Gutiérrez-Trejo, Matthew H. Van Dam, Athena W. Lam, Gonzalo Martínez-Herrera, Felipe A. Noguera, Thomas Weissling, Jessica L. Ware, Víctor H. Toledo-Hernández, Frederick W. Skillman Jr., Brian D. Farrell, Oscar Pérez-Flores, Lorenzo Prendini, James M. Carpenter

**Affiliations:** 1 Division of Invertebrate Zoology, American Museum of Natural History, New York City, NY USA; 2 Richard Gilder Graduate School, American Museum of Natural History, New York City, NY USA; 3 Entomology Department, Institute for Biodiversity Science and Sustainability, California Academy of Sciences, San Francisco, CA USA; 4 Center for Comparative Genomics, Institute for Biodiversity Science and Sustainability, California Academy of Sciences, San Francisco, CA USA; 5 The Graduate Center of the City University of New York, New York City, NY USA; 6 Estación de Biología Chamela, Instituto de Biología, Universidad Nacional Autónoma de México, San Patricio, JAL México; 7 Department of Entomology, University of Nebraska-Lincoln, Lincoln, NE USA; 8 Centro de Investigación en Biodiversidad y Conservación, Universidad Autónoma del Estado de Morelos, Cuernavaca, MOR México; 9 P. O. Box 375, Pearce, AZ USA; 10 Museum of Comparative Zoology, Department of Organismic and Evolutionary Biology, Harvard University, Cambridge, MA USA; 11 Laboratorio Nacional de Análisis y Síntesis Ecológica, Escuela Nacional de Estudios Superiores, Universidad Nacional Autónoma de México, Morelia, MICH Mexico

**Keywords:** Computational biology and bioinformatics, Ecology, Evolution, Systems biology, Zoology

## Abstract

*Tetraopes* longhorn beetles are known for their resistance to milkweed plant toxins and their coevolutionary dynamics with milkweed plants (*Asclepias*). This association is considered a textbook example of coevolution, in which each species of *Tetraopes* is specialized to feed on one or a few species of *Asclepias*. A major challenge to investigating coevolutionary hypotheses and conducting molecular ecology studies lies in the limited understanding of the evolutionary history and biogeographical patterns of *Tetraopes*. By integrating genomic, morphological, paleontological, and geographical data, we present a robust phylogeny of *Tetraopes* and their relatives, using three inference methods with varying subsets of data, encompassing 2–12 thousand UCE loci. We elucidate the diversification patterns of *Tetraopes* species across major biogeographical regions and their colonization of the American continent. Our findings suggest that the genus originated in Central America approximately 21 million years ago during the Miocene and diversified from the Mid-Miocene to the Pleistocene. These events coincided with intense geological activity in Central America. Additionally, independent colonization events in North America occurred from the Late Miocene to the early Pleistocene, potentially contributing to the early diversification of the group. Our data suggest that a common ancestor of Tetraopini migrated into North America, likely facilitated by North Atlantic land bridges, while closely related tribes diverged in Asia and Europe during the Paleocene. Establishing a robust and densely sampled phylogeny of *Tetraopes* beetles provides a foundation for investigating micro- and macroevolutionary phenomena, including clinal variation, coevolution, and detoxification mechanisms in this ecologically important group.

## Introduction

**Figure 1:**
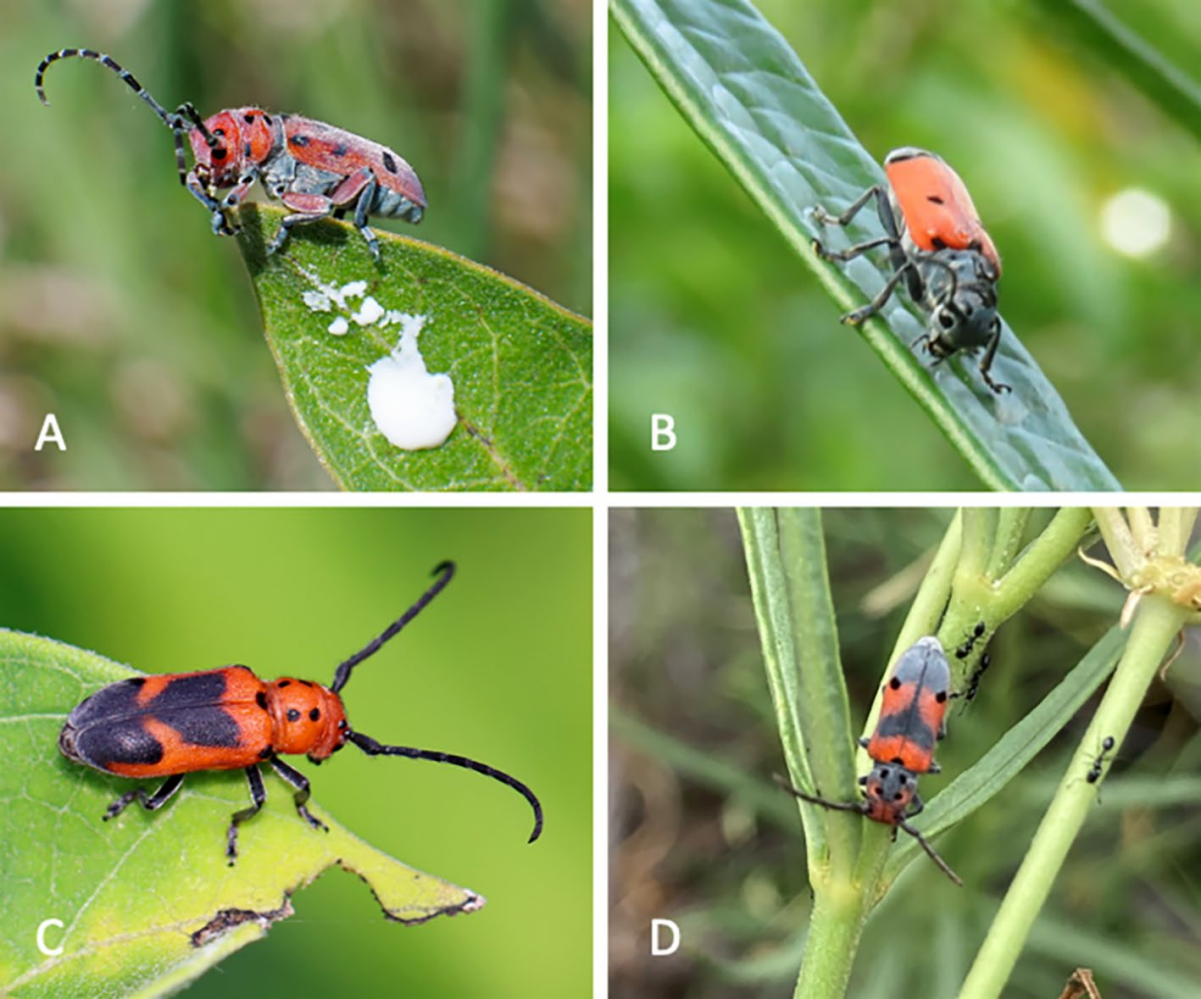
Adult *Tetraopes femoratus* (**A**), *T. cleroides* (**B**), *T. melanurus* (**C**), and *T. discoideus* (**D**). All photos from inaturalist. Photographers: Thomas Schultz, Gil Torres, Christine Young, and Miranda Kersten, respectively.

Beetles are the largest group of animals on earth, with more than 385,000 species described^1^. They have evolved an astonishing heterogeneity of trophic niches, behavior, and morphological diversity. Many beetles have developed very specialized life histories contributing to this diversity. For example, the Phytophaga beetle lineage is hyperdiverse and has members who often specialize on a single plant lineage^2,3^. One such example is the genus *Tetraopes* Dalman in Schönherr, 1817, a lineage of 26 species distributed in North and Central America (Fig. 1). This group is known as milkweed longhorn beetles because of their highly specialized feeding habits on plants of the genus *Asclepias* L. and other species of Apocynaceae Juss. plants^4^.

Studies on *Tetraopes* have thus far focused on addressing the gene mutations conferring resistance to milkweed’s toxins in the most common of its 26 species (*T. tetrophthalmus* Forster, 1771; throughout the document, ‘*T.*’ will refer to the genus *Tetraopes*) or assessing the coevolution of a subset of mainly North American species with its host plants^5–11^. The evolutionary history of *Tetraopes* remains poorly understood. One crucial but often overlooked component of coevolution is concordant biogeographical histories. Different biogeographical hypotheses have been proposed for the origin of the genus. Based on the distribution and species richness of *Asclepias* and *Tetraopes*, Linsley hypothesized that *Tetraopes* has southern affinities and is a descendant of the Sonoran fauna^12^. Later, Chemsak proposed the Alleghenian region as the center of origin of *Tetraopes*. Chemsak considered it more likely that *Tetraopes* derived from an Asian ancestor which colonized North America than to have originated in South America and later migrated into North America^4^. After mapping the distribution of *Tetraopes*, Farrell & Mitter (1998) proposed a tropical lowland origin for *Tetraopes* and subsequent colonization of temperate upland territories. Finally, Farrell suggested the mid-Tertiary as the time for contemporaneous diversification of both *Asclepias* and *Tetraopes*^11^. Nevertheless, the temporal and spatial changes of the genus have yet to be thoroughly investigated in a macroevolutionary framework.

There are several reasons to expect Central America (CA) to have played an important role in the diversification of *Tetraopes*. First, CA *Tetraopes* comprise 50% of the clade. Second, events of intense geological activity coincide with previously proposed times of origin of *Tetraopes* during the Neogene^11^. Third, the geological history of this region triggered the diversification of entire biomes and clades^13,14^. We hypothesize that adding Central American lineages to the analysis will help resolve deep and shallow nodes and discern biogeographical patterns within the genus.

We employ state-of-the-art methods to address the evolutionary history of *Tetraopes* beetles and their closest relatives by integrating genomic, morphological, paleontological, and geographical data. We emphasize sampling of CA species, where most of the species of the genus are distributed. Thus, our goals are: (1) to generate a phylogeny of *Tetraopes* and evaluate node stability through a sensitivity analysis; (2) employ paleontological evidence to date the divergence of *Tetraopes* and closely related lineages; and (3) address the biogeographical history at a local scale including *Tetraopes* species divergences, as well as in a broader scale by studying species from closely related tribes to understand patterns in deep time.

## Results

### Genome assembly and UCE capture

The assembly of *T. tetrophthalmus* comprised 771 Mbp distributed across 61,094 scaffolds with an N50 of 30,733 bp. BUSCO analyses found 88.1% complete genes, with 84.6% representing complete and single-copy genes, 3.5% complete and duplicated, 6.6% fragmented, and 5.3% missing. The remaining genome assemblies ranged in size from 36 to 715 Mb. Gene completeness was very dissimilar among species, ranging from 3 to 88%. GC content ranged from 30 to 55%. N50 values ranged from 669 to 7276 (Supplementary Table 3).

The custom Lamiinae probe set selected 17,086 loci among the eight taxa included in the design, with an average of 10,855 UCEs (ranging from 512 to 13,081) recovered from the 36 species. We confirmed previous findings on the decreasing numbers of loci captured in relation to increasing phylogenetic distance in insects, using the Coleoptera probe set 1.1Kv1^15–17^. To enhance capture success, a more comprehensive species sampling of the focal taxon seems necessary. We found that specimen preservation method affected both UCE number and length, with strategies such as obtaining extra sequences for dry pinned samples potentially improving outcomes, albeit mainly in mean length quality.

### Phylogenomics

**Figure 2:**
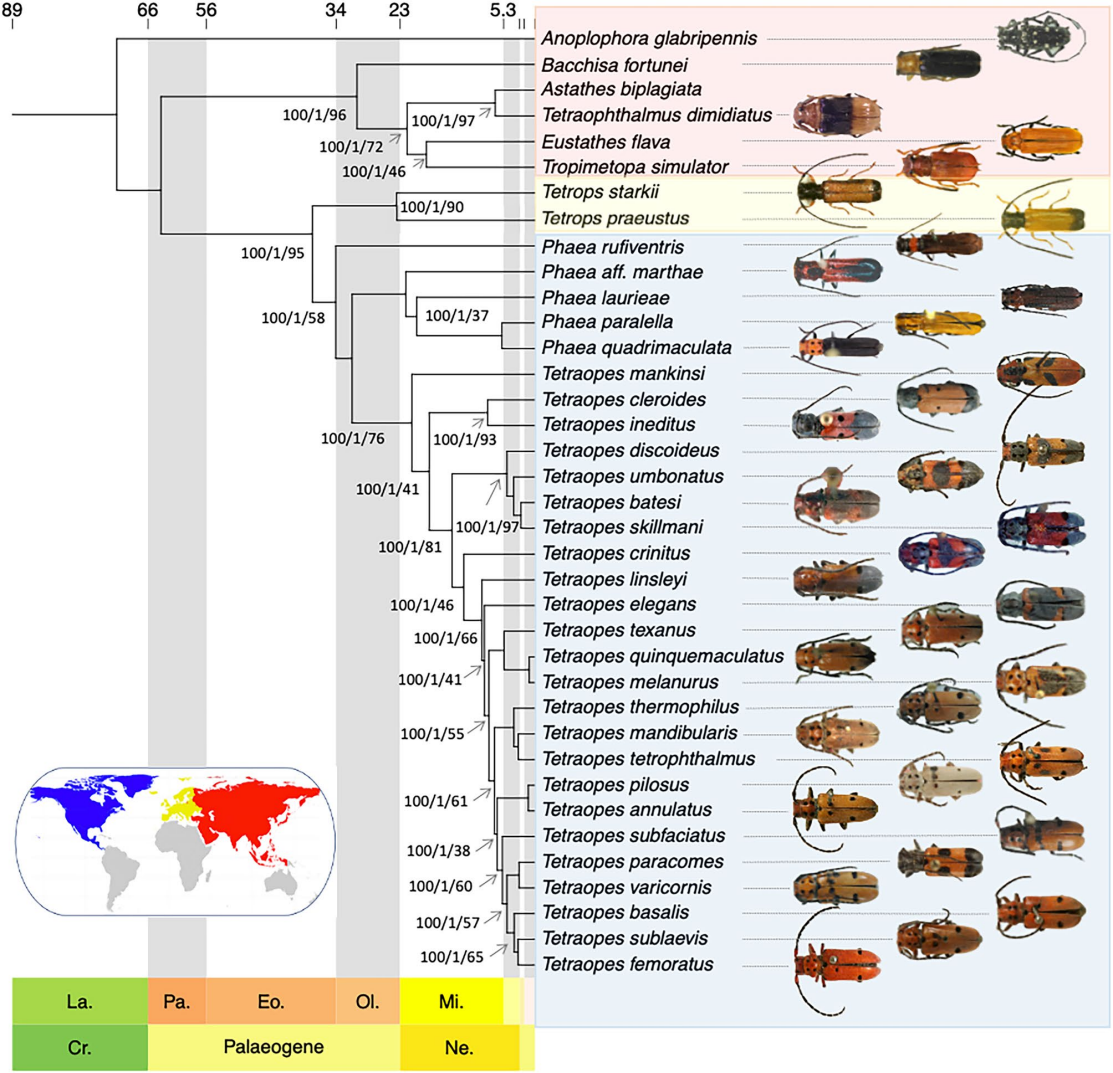
Dated phylogeny of *Tetraopes* and relatives. Support values from Maximum Likelihood, Bayesian Inference, and Coalescence are indicated on each node as Bootstrap (0–100)/MP (0–1)/NQS (0–100). Colors indicate the geographical distribution of species in the map. Image source:^19^. The map was generated in R (version 4.3.2; https://www.r-project.org), with ggplot2 (https://ggplot2.tidyverse.org) using freely accessible vector map 1:50 m data (https://www.naturalearthdata.com) without requiring any permission from external sources.

We analyzed three UCE matrices varying in loci number after filtering by completeness and by PIS (Table 1): a 50% complete matrix (12,158 loci), 75% complete matrix (10,025 loci), and 90% complete matrix (2859 loci). The nine topologies obtained from different completeness matrices and three phylogenetic inference methods, recovered the same phylogeny, with three major monophyletic lineages: Astathini, Tetropini, and Tetraopini, the latter with genus *Phaea* placed sister to *Tetraopes*. All analyses recovered *Tetraopes* as monophyletic with high support (Fig. 2, Table 2). Furthermore, all topologies recovered *Phaea mankinsi* within *Tetraopes,* being the basalmost species of the clade. *T. ineditus* Chemsak & Giesbert, 1986 and *T. cleroides* Thomson, 1860 formed a clade sister to the rest of the species. *T. discoideus* LeConte, 1858*, T. umbonatus* LeConte, 1852, *T. skillmani* Chemsak & Noguera, 2004, and *T. batesi* Chemsak, 1963 formed a clade, also recovered in the morphological tree described in the next section. The next species to diverge are *T. crinitus* Chemsak & Noguera, 2004*, T. linsleyi* Chemsak, 1963, and *T. elegans* Horn, 1894, followed by the clade comprising *T. texanus* Horn, 1878, *T. melanurus* Schönherr, 1817, and *T. quinquemaculatus* Haldeman, 1847. In the sister clade, the first groups to diverge were *T. thermophilus* Chevrolat, 1861, *T. tetrophthalmus*, and *T. mandibularis* Chemsak, 1963, and the clade comprising *T. annulatus* and *T. pilosus*. Next, *T. subfasciatus* Bates, 1881 diverged as the sister to the remaining species, distributed in two main groups; *T. varicornis* Castelnau, 1840 and *T. paracomes* Chemsak, 1963; and *T. basalis* LeConte, 1852, *T. femoratus* LeConte, 1847, and *T. sublaevis*.

**Table 1:** Number of loci after filtering by completeness and by parsimony informative sites (PIS).

Filtering criteria	50%	75%	90%
By completeness	13,137	11,700	3329
By PIS	12,158	10,025	2859

**Table 2:** Sensitivity analysis indicating clade support for each subset of data and inference method.

Clade	Maximum likelihood			Bayesian inference			Coalescence		
	Bootstrap			MP			NQS		
	50%	75%	90%	50%	75%	90%	50%	75%	90%
*Tetraopes* (*P. mankinsi* + rest of *Tetraopes* spp.)	100	100	100	1	1	1	76.78	77.31	76.27
*T. ineditus, T. cleroides*	100	100	100	1	1	1	94.21	93.70	92.96
(*T. crinitus* (*T. discoideus* (*T. umbonatus* (*T. batesi. T. skillmani*)	100	100	100	1	1	1	NA	NA	NA
(*T. discoideus* (*T. umbonatus* (*T. batesi. T. skillmani*)	NA	NA	NA	NA	NA	NA	97.21	96.93	96.82
(*T. crinitus* (*T. linsleyi (T. elegans* (*T. texanus* (*T. melanurus. T. quinquemaculatus*)	NA	NA	NA	NA	NA	NA	47.26	47.54	46.40
(*T. linsleyi* (*T. elegans* (*T. texanus* (*T. melanurus. T. quinquemaculatus*)	100	100	100	1	1	1	NA	NA	NA
(*T. thermophilus* (*T. tetrophthalmus. T. mandibularis*)	100	100	100	1	1	1	95.02	94.75	94.87
*T. annulatus*, *T. pilosus*	100	100	100	1	1	1	98.45	98.34	98.35
(*T. subfaciatus* (*T. varicornis*, *T. paracomes*) (*T. basalis* (*T. femoratus*, *T. sublaevis*)	100	100	100	1	1	1	61.26	60.42	59.96
*Phaea* (*P*. aff. *marthae* (*P. lauriae* (*P. parallela*, *P. quadrimaculata*)	100	100	100	1	1	1	82.96	83.69	84.31
Astathini (*B. fortunei* (*E. flava, T. simulator*) (*A. biplagiata. T. dimidiatus*)))	100	100	100	1	1	1	97.10	96.14	96.45
Tetropini (*Tetrops starkii, Tetrops praeustrus*)	100	100	100	1	1	1	89.21	89.75	89.66

The only conflict in topologies was the position of *T. crinitus* which could either be sister to all other species in the largest *Tetraopes* clade or part of the clade comprising *T. discoideus, T. umbonatus, T. skillmani*, and *T. batesi*. The first option was strongly supported by coalescent-based analysis with over 90% agreement from gene trees in the three matrices, while the alternative topology was better supported by ML and BI analyses (Table 2).

The ML analyses in RAxML resulted in topologies with 100% support for all clades in all three matrices. Bayesian Inference in Exabayes received good convergence statistics with ASDSF values < 1%, ESS values > 200, and PSRF values < 1.1. The three topologies from different completeness matrices were identical with a marginal probability of 1 in all nodes. Multi-species coalescent phylogenies had an LPP of 1 in all nodes, except for an internal *Tetraopes* node, and a group sister to the clade of *T. discoideus, T. umbonatus, T. skillmani*, and *T. batesi*. The final normalized quartet score indicated low discordance in the gene trees, ranging from 90.6 to 90.7, and normalized quartet support (NQS) varied from 36–37 to 99% across completeness matrices.

### Divergence‐time estimate analysis

The parsimony analysis resulted in a single tree with 126 steps. *Tetrops rottensis* and *Tetrops praeustus* formed a clade sister to all Tetraopini species. The morphology-based phylogeny included two major *Tetraopes* clades: one comprised *T. discoideus, T. skillmani, T. umbonatus*, and *T. batesi*, while the other comprised *T. tetrophthalmus, T. femoratus, T. annulatus*, and *T. pilosus* Chemsak, 1963. The position of *Phaea* as the sister group of *Tetraopes* was also confirmed. Convergence diagnostics confirmed that independent runs in MCMCTree reached convergence, and a comparison of prior and posterior densities of node ages from the independent rate model revealed no substantial truncation effects.

### Ancestral range estimations

Ancestral range estimation in BioGeoBEARS at a global geographical scale recovered DEC + J as the best-fit model to explain the diversification of Astathini, Tetraopini, and Tetropini clades (LnL = − 40.01, AICc = 86.03) (Table 3). Furthermore, the analysis focused on *Tetraopes* species recovered BAYAREALIKE + J model as the most appropriate for the dataset (LnL = − 110.15, AICc = 167.5) (Table 4).

**Table 3:** Results for the six biogeographical models tested in BioGeoBEARS at global geographical scale.

Model	LnL	Parameters	d	e	j	AICc	AIC_wt
DEC	− 52.01	2	0.0110	4.4e−03	0.000	108.00	1.1e−05
DEC+J	− 40.01	3	0.0037	0.0e+00	0.027	86.03	6.7e−01
DIVALIKE	− 54.62	2	0.0150	5.0e−03	0.000	113.20	8.0e−07
DIVALIKE+J	− 41.09	3	0.0040	0.0e+00	0.029	88.18	2.3e−01
BAYAREALIKE	− 65.77	2	0.0130	2.7e−02	0.000	135.50	0.0e+00
BAYAREALIKE+J	− 41.92	3	0.0033	1.0e−07	0.032	89.84	1.0e−01

Best-fit models are highlighted.

**Table 4:** Results for the six biogeographical models tested in BioGeoBEARS for Tetraopes only analysis.

Model	LnL	Parameters	d	e	j	AICc	AIC_wt
DEC	− 77.32	2	0.0470	2.8e−02	0.000	158.6	0.0020
DEC+J	− 75.29	3	0.0340	1.1e−02	0.070	156.6	0.0055
DIVALIKE	− 78.34	2	0.0580	2.9e−02	0.000	160.7	0.0007
DIVALIKE+J	− 77.42	3	0.0460	1.8e−02	0.039	160.8	0.0007
BAYAREALIKE	− 80.19	2	0.0700	1.4e−01	0.000	164.4	0.0001
BAYAREALIKE+J	− 70.10	3	0.0094	1.0e−07	0.110	146.2	0.9900

Best-fit models are highlighted.

**Figure 3:**
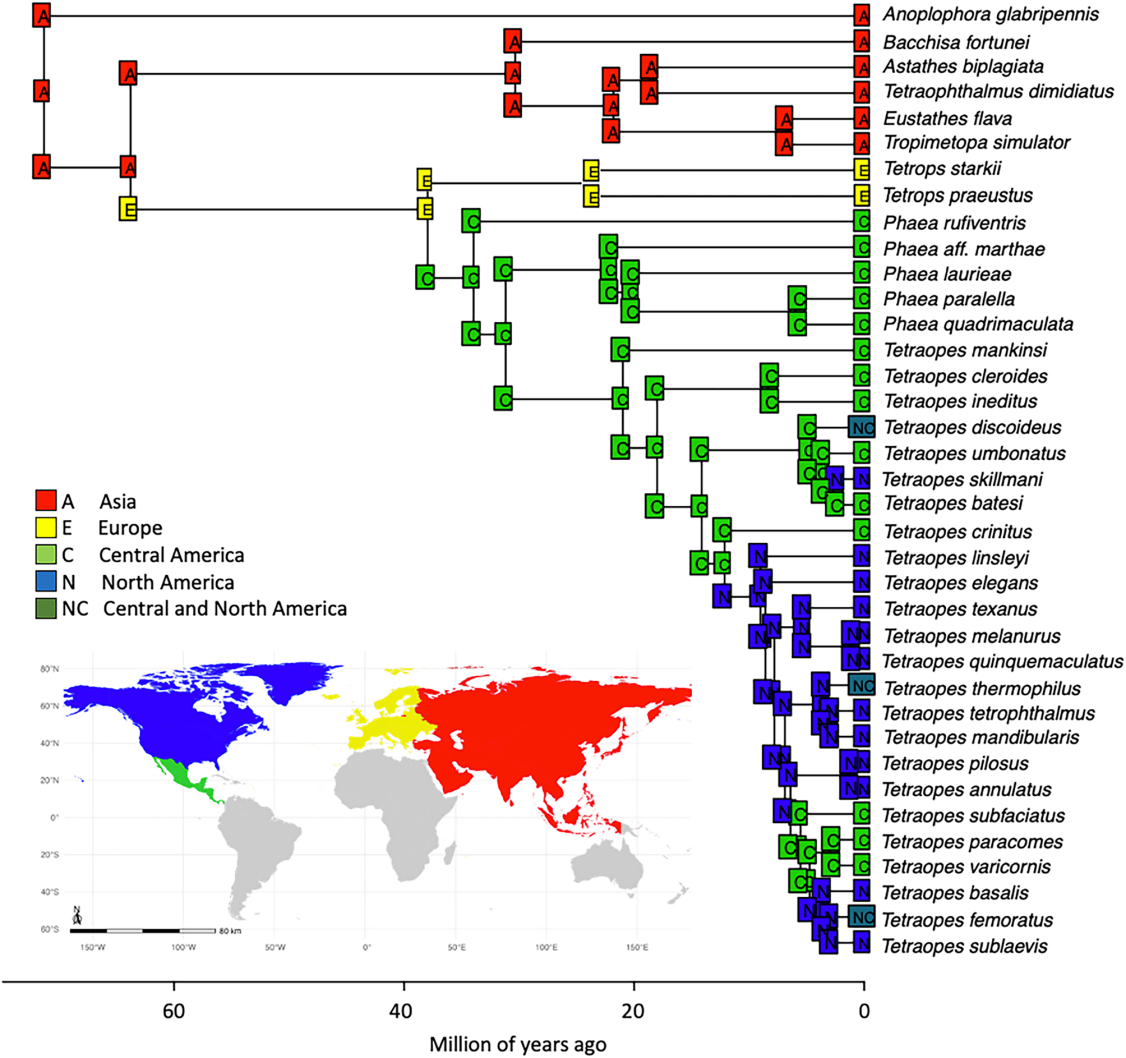
BioGeoBEARS best-fit model (DEC+J) for the tribes Astathini, and Tetropini, and Tetraopini. The map was generated in R (version 4.3.2; https://www.r-project.org), with ggplot2 (https://ggplot2.tidyverse.org) using freely accessible vector map 1:50 m data (https://www.naturalearthdata.com) without requiring any permission from external sources.

The time-calibrated phylogeny estimated the origin of tribe Astathini at 33 mya (HPD 21–38 Ma), and its ancestor was estimated to have occurred in Asia. The following lineage to diverge was the tribe Tetropini 24 mya (HPD 23–24 Ma) in the late Oligocene, with Europe as the ancestral geographical range inferred with the highest probability. Tribe Tetraopini, which comprises the genera *Phaea* and *Tetraopes,* was estimated to have diverged in the early Oligocene, around 34 mya (HPD 28–40 Ma), and the most likely ancestral range of this clade is Central America (Fig. 3).

**Figure 4:**
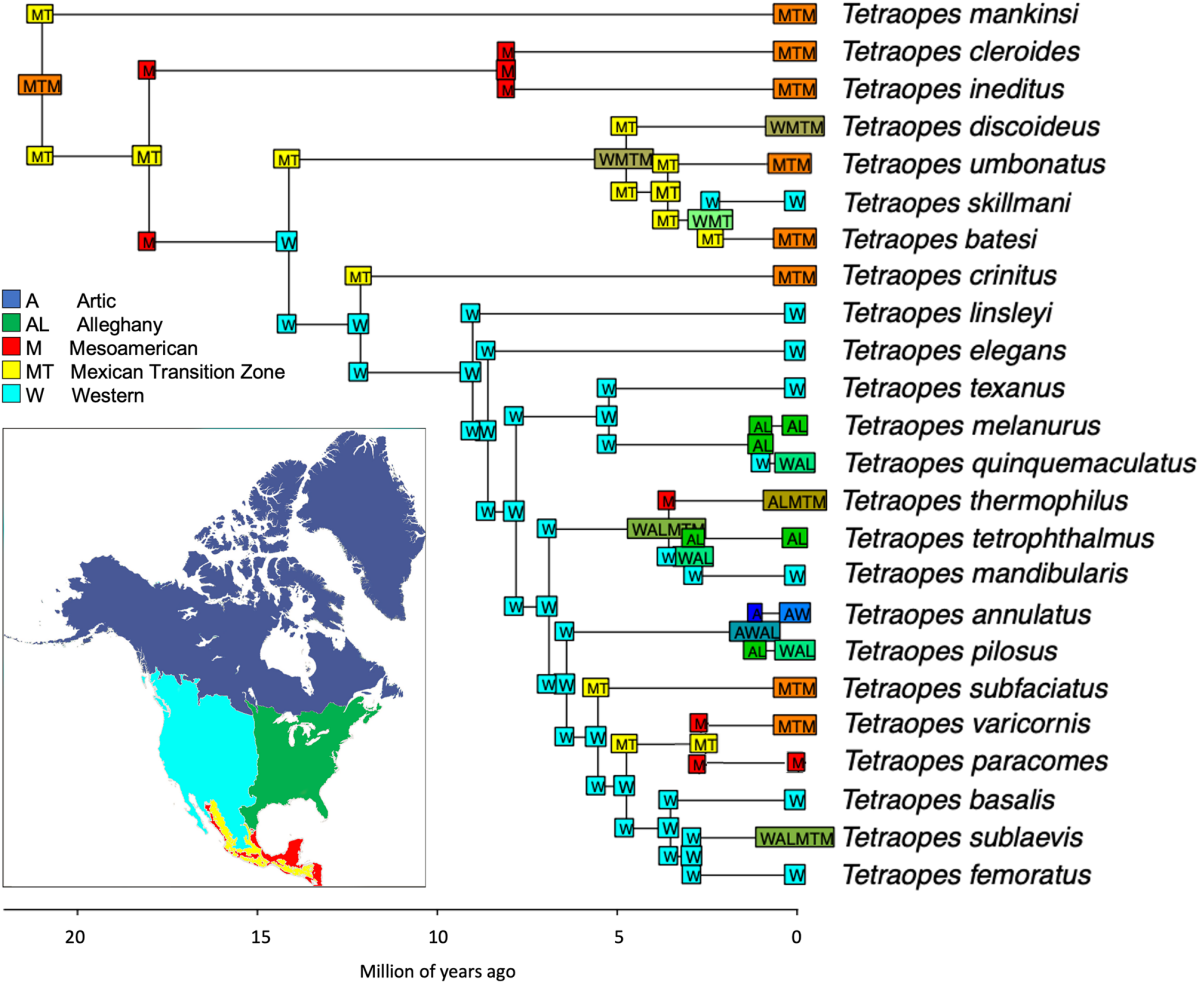
Map summarizing (**A**) the inferred colonization scenario for the tribes Astathini, and Tetropini, and Tetraopini. North Atlantic land bridges are shown as the most likely dispersal route of the ancestor of Tetraopini. Map was modified in GIMP (version 2.10; https://www.gimp.org) from^84^, published under the Creative Commons Attribution- NonCommercial 4.0 International License (CC BY-NC) open-access license without requiring any permission from external sources.

The crown group of *Tetraopes* was estimated to have originated in the last ~ 21 Ma (HPD 17.1–24 Ma) during the late Oligocene and early Miocene. The Mexican Transition Zone and Mesoamerica (MTZ-M) were estimated as the ancestral geographical range for the clade with the highest probability. *Tetraopes mankinsi*, *T. ineditus,* and *T. cleroides,* which diverged 21 mya (HPD 17.1–24), and 8 mya (HPD 4.8–11.4), retained a MTZ-M distribution. Subsequent northward dispersal by the remaining *Tetraopes* clades along with the crown group diversification during the Miocene and Pleistocene. The largest *Tetraopes* clade with 17 species diverged ~ 12 mya (HPD 9.6–14.6) and is represented by a mixture of biogeographic lineages, which include areas such as the Mesoamerican, Alleghany, and the Arctic (Fig. 4).

## Discussion

*Tetraopes* comprises 26 species distributed from southern Canada, throughout the United States, as far south as Costa Rica^18,19^. The taxonomic history of *Tetraopes* begins with its description by Dalman in an unpublished work, which Schönherr later published in 1817^20^. Guérin-Méneville (1844) designated *Tetraopes tetrophthalmus*^21^ as the type species of the genus^4^. After that, the most prolific period of new species discovery was the nineteenth century, in which 58% of the 26 species currently known, were described. In the twentieth century, 27% of the species were described whereas, in the twenty-first century, 13% were described^18^. The first revision of the genus by Casey in 1913 included 28 species described at that time (not all valid today)^22^. The most comprehensive morphological study of *Tetraopes* to date was published by Chemsak in 1963^4^. Chemsak reviewed the taxonomy and ecology of the 22 species recognized at the time, providing a key to the adults, along with geographical distribution maps and detailed illustrations.

Thirty-five years after Chemsak’s revision, an attempt to analyze possible coevolutionary diversification in *Asclepias* and *Tetraopes* generated the first phylogeny of the genus based on allozyme data for 13 species^10^. A parsimony analysis of the data supported the monophyly of the genus, although it had several weakly resolved nodes. In a subsequent study, cytochrome oxidase I (COI) sequences of the species analyzed previously were used to establish a molecular clock that estimated a genus age of 15 million years^11^. Both phylogenetic analyses of *Tetraopes* included only 50% of the species of the genus and were based on only a limited number of characters. Recent taxonomic changes in *Tetraopes* species include the transfer of *T*. *mankinsi* Chemsak & Linsley, 1979 to the genus *Phaea* Newman, 1840, apparently because it did not match the diagnosis of *Tetraopes*, although few details were provided^23^. The species was initially described as *T*. *mankinsi* in 1979 based on specimens from Honduras and El Salvador^24^. Also, *T. huetheri* was described and subsequently synonymized with *T. annulatus* LeConte, 1847^25,26^.

Since previous molecular studies only sampled approximately 50% of the genus, comparing previous topologies to our more complete sampling effort is only partially instructive. Phylogenetic relationships of *Tetraopes* based on fewer taxa and characters^10,11^ were only partially confirmed by our results. Previous topologies differed substantially in the most recently divergent clade. Similarly to the previous hypotheses, our results recovered *T. discoideus* and *T. umbonatus*, and *T. texanus*, *T. melanurus*, and *T. quinquemaculatus* as closely related species. Relationships between the *T. annulatus* + *T. pilosus* and *T. tetrophthalmus* + *T. mandibularis* as sister groups were also confirmed by this work. Interestingly, previous phylogenetic hypotheses based on allozymes^10^, COI^11^, and our results with over 12 thousand UCE loci obtained different combinations for one of the most recently divergent clades, which includes *T. sublaevis, T. femoratus*, *T. varicornis,* and *T*. *basalis*. The major discrepancy concerned the position of *T. basalis*, recovered as sister to *T. sublaevis*^10^, to *T. varicornis*, *T. femoratus*, and *T. varicornis*^11^, and to *T. sublaevis* and *T. femoratus* (this work). This clade also includes *T. paracomes* in our analyses*.*

In addition to conflicting topologies, the COI analyses yielded differing divergence time estimations compared to this work. According to our results, the crown age of *Tetraopes* is older than estimated based on COI (21 and 15 mya, respectively)^11^. However, the ages of divergence of internal nodes are, in some cases, younger than previously proposed^11^. This incongruence in age estimates is likely due to differences in the datasets (300 loci alignment vs. mtDNA-only), taxon sampling (36 species vs. 13), and calibration strategies. More taxa and data resolved the relationships and provided more precise divergence time estimations.

Regarding the taxonomic changes based on the phylogenetic analyses, *Phaea mankinsi* appears to belong to *Tetraopes*. The stability of the node in which *P. mankinsi* was placed within *Tetraopes* was tested with a sensitivity analysis that explored how parameters affect phylogenetic hypotheses^27^. This node has maximum bootstrap and marginal support from ML and Bayesian analyses and 76-77 of NQS from a coalescent-based study (Fig. 2). Also, the DNA of the species used in this work was obtained from the holotype. Here, we provide evidence to transfer *P. mankinsi* to *Tetraopes*: *Tetraopes mankinsi* Chemsak & Linsley, 1979, new status.

With respect to tribe classification of Tetraopini, the tribe containing *Tetraopes*, the most recent phylogeny of Lamiinae recovered Tetraopini, Tetropini, and Astathini as clades in analyses with Maximum Likelihood (ML) and Bayesian inference with high support values^28^. The need to redefine the classification of the three tribes has been recognized, as their morphology is rather uniform and they have been separated based mainly on their geographical distributions (Astathini in Asia, Tetropini in Europe, and Tetraopini in the Americas)^28^. In this work, we studied the type genera of the three tribes (*Tetrophthalmus*, *Tetrops*, and *Tetraopes*) and confirmed their monophyly^29^. In addition, this represents the first biogeographical analysis to shed light on how and when these tribes diverged to archive their current distributions. Our results provide robust support for synonymizing two of the tribes into the nominotypical tribe, which would be the subject of future study.

The body of knowledge about *Tetraopes* beetles has accumulated over two hundred years. This has included knowledge about their taxonomy and distribution, and in particular, has focused on understanding its conspicuous relationship with host plants of the genus *Asclepias*. However, a key piece to integrating all this information had been missing, a comprehensive analysis of their phylogenetic relationships and biogeographical history.

Here, we present a phylogeny and biogeographical history of 23 species of the genus *Tetraopes* and their closely related genera. On a broader geographical scale, ancestral range estimations of the tribes Astathini, Tetropini, and Tetraopini suggest the occurrence of at least two founder-event speciations during the evolutionary history of the tribes (Fig. 3). According to Matzke^30^, founder events imply long-distance colonization that founds a population genetically isolated from the ancestral population. The two founder events in the history of the three tribes are: (1) Tetropini species colonizing Europe from Asia during the Late Oligocene/Early Miocene; (2) Tetraopini ancestor colonizing North America from Europe during the Late Eocene and Early Oligocene. Our dating analyses estimated divergence times of the later colonization event (34 Ma, HPD 28–40 Ma) correspond well with the onset of the Bering Land Bridges (BLB) and the North Atlantic land bridges (NALB)^31^.

Although our estimation of divergence times suggest that the ancestor of Tetraopini may have colonized North America from Europe both via BLB and NALB, the most parsimonious biogeographical scenario considering the present-day occurrence of *Tetrops* and the fossil record is that the ancestor of Tetraopini dispersed from Europe to North America via NALB. Only one of the 17 species in the genus is distributed in Asia, with most of the diversity concentrated in South and Central Europe^19,32^. Also, the fossil species *Tetrops rottensis* was described as part of the insects found in the Rott lagerstatten in Germany, which implies an ancient occurrence of early-divergent lineages of *Tetrops* in Europe, supporting an early NALB migration route rather than the BLB^33^.

Our results suggest an origin of *Tetraopes* ~ 21 mya during the Miocene (probably late Oligocene, considering the wide 95 HPD of the divergence times of the node). The Mesoamerican and Mexican Transition Zone were recognized as the ancestral ranges of the clade (Fig. 4). Both regions experienced intense geological activity during that period, with the formation of the mountain systems of Mexico and Central America from the mid-Miocene to the Pliocene. As a result, new habitats and climatic conditions were generated and impacted the diversification of species in the region, as reported for other taxa^13,14^. One of the biomes that could have been produced by the formation of the Sierra Madre Occidental and the Neovolcanic belt is the dry forest, where several *Tetraopes* species are distributed. Thus, rapid cladogenesis of *Tetraopes* species could have been triggered by the establishment of modern biomes in western and central Mexico, resulting from the formation of mountain systems in the region^34–36^.

After the origin of the major clades of *Tetraopes* in the Mesoamerican and Mexican Transition Zone, there were at least two independent colonization events in the Western and Alleghany areas. One occurred in the late-Miocene ~ 9 mya in the lineage leading to the largest *Tetraopes* clade, currently including 16 species. This colonization coincides with a global decrease in temperature and humidity during the Late Miocene, which gave rise to postglacial dispersal northward in some taxa^37–39^. The second colonization event to northern Mexico and the southwestern US involved a lineage that gave rise to *T. skillmani*. This colonization constitutes one of only three divergences during the Pleistocene (*T. batesi* and *T. skillmani*; *T. annulatus* and *T. pilosus*; *T.quinquemaculatus* and *T. melanurus*). During this time, desert formation in North America started in the Miocene and continued into the Pleistocene, as well as Holocene climatic shifts, which connected and disconnected the eastern and western deserts of North America^40,41^. Therefore, the current distribution of *Tetraopes* species results from colonization of its northern range from southern regions in the vicinity of the MTZ and the M areas.

The existence of a robust and densely sampled phylogeny of *Tetraopes*, along with an exploration of its diversification across major biogeographical regions and biomes, will significantly enhance our understanding of evolutionary processes, including coevolution and insect-plant interactions. Furthermore, it provides a framework for comprehending micro- and macroevolutionary processes, such as clinal variation, speciation, and diversification.

## Methods

### Specimen collection

A total of 36 specimens were used in this study, including 23 species of *Tetraopes* and 13 species from the Tetropini and Astathini tribes. Adult specimens were collected from Mexico and the United States. Samples were preserved in liquid nitrogen or in 98% ethanol and stored at − 70 °C. Specimens from the Czech Republic, Slovakia, Indonesia, the Philippines, and Japan were also included.

Specimens from entomological collections were assessed for molecular and morphological work. Specimens were identified using taxonomic keys^4,23^, and the identity of non-American species was corroborated by experts (Petr Švácha, Academy of Sciences of the Czech Republic; Karl Adlbauer, Austria; and Junsuke Yamasako Japanese Institute for Plant Protection). For a complete list of specimens and collecting localities, see Supplementary Table 1.

### DNA extraction and library preparation

DNA was extracted from body tissue and legs; legs were punctured to facilitate the action of proteinase-k. MagAttract HMW DNA Kit was used to isolate high molecular weight genomic DNA from *T. tetrophthalmus* following the manufacturer’s protocol for solid frozen tissue and recommendations from 10× Chromium DNA Extraction from Single Insects^42^. OmniPrep DNA Extraction kit, DNeasy Blood & Tissue Kit, and Qlamp mini kit were used for DNA extractions of other specimens. DNA fragment size was quantified using Qbit 2.0 fluorometric quantification (Invitrogen, USA), a Bioanalyzer, and 0.5% agarose gel electrophoresis. In some cases, DNA was sheared before library preparation in a Covaris M220 (Covaris Inc., USA).

After DNA extraction, samples were divided into two groups for library preparation and sequencing. The first included 30 specimens for which library preparation was performed at the Center for Comparative Genomics of the California Academy of Sciences. Library preparation for these samples was conducted using NEBNext Ultra II DNA Library Preparation kit (New England Biolabs Inc, USA) following the manufacturer’s protocol (size selection protocol for fresh samples, and without size-selection for degraded DNA extracted from museum samples) and later sequenced in Illumina Novaseq, 150 bp paired-end reads. The second group included six samples for which an external company performed library preparation. A 10× Genomics Chromium linked-read library was prepared for *T. tetrophthalmus*, whereas standard Illumina libraries were generated for the other samples. Libraries were sequenced on Illumina HiSequ, 2 × 250 base-pairs (bp), and generated linked reads for *T. tetrophthalmus* and short paired-end Illumina reads for the other species (Supplementary Table 2). Reads were subject to quality control on Fastp 0.23.2^43^ and removal of Illumina universal adapters.

### Genome assembly

The genome of *T. tetraophthalmus* was assembled in Supernova 2.1^44^ with default settings. Remaining genomes were assembled using SPAdes 3.15^45–47^ with k-mer values of 21, 33, 55, 77, 99, and 127, as recommended for read lengths of 150 bp.

### Ultraconserved Element (UCE) custom probe set design

An in-silico test of the Coleoptera UCE probe set version 1.1Kv1 was performed on six *Tetraopes* genomes with the PHYLUCE tutorial III^16,48^. The probe set captured only 380–390 (~ 33%) of the 1172 UCEs in the Coleoptera set. However, as 17 museum specimens were to be included in the sampling (some of them over 70 years old), there was the possibility of the number of UCEs captured to be even lower because fragmented DNA decreases the performance of the probes^17,49^.

As UCE probe sets customized for a focal group have resulted in a larger number of recovered loci in other insect groups^15,50–52^, we designed a customized set of probes for Lamiinae using the PHYLUCE 1.7.1 pipeline^48^. Eight species were included in the design (7 Lamiinae and one Cerambycinae), including *T. tetrophthalmus* (generated in this work), *Anoplophora glabripennis* [^53^, Agla_2.0], *Doliops geometrica* Waterhouse, 1842 (Van Dam, unpublished data), *Aprophata* aff. *notha* (Newman, 1842) (Van Dam, unpublished data), *Achriotypa basalis* Pascoe, 1875 (NHI Accession No. SRR15249232), *Similosodus venosus* (Pascoe, 1867) (NHI Accession No. SRR15249233), *Rhytiphora diva* (Thomson, 1860) (NHI Accession No. SRR15249221), and *Turanoclytus namaganensis* (Heyden, 1885) (NHI Accession No. SRR16700842). The species used as a base taxon was *A. glabripennis* because it corresponds to the same subfamily as *Tetraopes* beetles. At the time of the study, it was the most complete genome available for the group. The other species belong to four Lamiinae tribes spanning the phylogenetic diversity of the subfamily Lamiinae (Apomecynini, Lamiini, Tetraopini, and Pteropliini)^28^. Soft masked files were used following guidelines of probe design^50,54^.

### UCE matrix generation and partitioning

We used the PHYLUCE^48^ pipeline with default settings to extract probes from the assembled genomes. After aligning with MAFFT^55^ and trimming the conserved locus matrices, we filtered for completeness by generating concatenated matrices in which the loci retained at least 50, 75, and 90% of the taxa. We conducted additional filtering on each completeness matrix by calculating the number of parsimony-informative sites (PIS) using a script implemented in Phyloch^56,57^. Informed by an examination of the loci distribution and their associated PIS, we established a threshold to retain loci with 50 to 250 PIS (50 < PIS < 250). Loci with lower and higher PIS were considered low-informative and highly-informative outliers, respectively, often associated with increased phylogenetic noise for topological inference and saturation in studies across different taxa^58–60^.

Each matrix was subsequently input to the Sliding-Window Site Characteristics (SWSC) method that accounts for UCE heterogeneity and increases the model fit^61^. Following this step, for each matrix, we used PartitionFinder2^62^ to find the best-fit models for the subsets created by SWSC with the following settings: linked branch lengths, GTR, GTR+G, GTR+I+G models, AICc as criteria for model selection, and rclusterf search.

### Phylogenomics

Phylogenetic analyses were performed on each completeness matrix (50, 75, and 90%) with different phylogenetic inference methods (Maximum Likelihood, Bayesian Inference, and coalescent-based analysis). For Maximum Likelihood analysis, RAxML-NG v. 0.8.0^63^ with default settings was used, confirming convergence with the default cutoff for large data sets and mapping bootstrap values onto the best ML tree.

Bayesian inference in ExaBayes 1.5.1^64^ was performed by two independent Markov chain Monte Carlo (MCMC) searches, each with one cold and one heated chain, with default parameters, a 25% burn-in, and linking all partitions into a single branch length parameter. Non-partitioned data were used for these analyses. The Average Standard Deviation of Split Frequency (ASDSF) was used to evaluate convergence, with ESS and PSRF values also examined. A consensus tree was generated from sets of trees.

For the multi-species coalescent approach, gene trees were generated with IQ-TREE 2.0.3^65^ ultrafast bootstrap with 1000 replicates for ASTRAL 5.7.8^66^ input. ASTRAL was run with default settings, and support values, local posterior probabilities (LPP), and normalized quartet support (NQS) values were mapped in the tree.

### Divergence time estimation

The fossil *Palaeoncoderes piacentinii* (59.2 Ma) was used as minimum bound as it is the most recent Lamiinae fossil described^67^. *Cretoprionus liutiaogouensis*, the earliest known cerambycid beetle, served as the maximum bound for the root (124 Ma)^68^.

The fossil species *Tetrops rottensis*^33,69^ was used as a prior to time-calibrate the Tetropini clade. Its phylogenetic position was unconfirmed, so the species was first included in a phylogenetic analysis to confirm that it would be an appropriate calibration point^70^. A morphological matrix was generated, including *Tetrops rottensis*, *Tetrops praeustus* Linné, 1758, eigth *Tetraopes* species, one *Phaea* species, and *Anoplophora glabripennis* as outgroup.

The morphological matrix included 50 characters and 12 taxa (Supplementary Table 1 and Supplementary List 2). Observations and measurements of the specimens were conducted using a Nikon SMZ 1500 microscope. The ‘body length’ character was coded as continuous and standardized in TNT 1.6 with the nstates stand command. A parsimony analysis was conducted in TNT using new technology search strategies (10 random seeds, find minimum length 20 times, and trees collapsed after search). Symmetric resampling and bootstrap support were performed with a removal probability of (p) 1⁄4 33, 1000 pseudoreplicates, collapsing the nodes below P 1⁄4 50^71–73^.

After confirming the phylogenetic position of *Tetrops rottensis*, normal age distributions were generated in MCMCtreeR^74^ (root: 59.2–124 mya, Tetropini node: 24.2–23 mya) to account for the uncertainty of the fossil record. Input files for MCMCTree^75^ consisted of a MAFFT^55^ alignment of the 300 most PIS loci and the ASTRAL phylogeny as the starting topology. The same data was employed to estimate the substitution rate in baseml^75^.

Using a relaxed-clock model with independent distributed rates, branch lengths, gradient, and Hessian were estimated, followed by the estimation of divergence times^76^. Sampling included a burn-in of 50,000 generations followed by 500,000 posterior samples every 50 generations. A replica with random seeds was also run. Convergence diagnostics were calculated, including comparing the posterior means among the different runs to evaluate whether they converged and generating several convergence statistics (posterior mean, ESS, posterior variance, and standard error of posterior means)^77^. Finally, prior densities of node ages were compared to posterior densities after sampling from the prior with a replica and convergence evaluation.

### Ancestral range estimation

The R package BioGeoBEARS^78^ was employed for ancestral range estimation of the time-calibrated phylogeny, which served as a fixed topology. Presence-absence matrices were generated based on geographical records from two Cerambycidae databases^18,19^. The analysis included an areas adjacent matrix indicating adjacent (1) and nonadjacent (0) areas. Six models of evolution were evaluated (1) Dispersal-Extinction-Cladogenesis (DEC)^79^, (2) DEC+founder-event speciation (“jump”; DEC+J), (3)Dispersal-Vicariance Analyses (DIVALIKE)^80^, (4) DIVALIKE+J, (5) Bayesian inference of historical biogeography for discrete areas (BAYAREALIKE)^81^, and (6) BAYAREALIKE+J. The Akaike Information Criterion (AICc)^82^ was used to determine the likelihood of the dataset given each model.

Two geographical scales were used, global with all the species and the American scale focusing on *Tetraopes* species. For the former, four biogeographical areas were defined, based on previous studies, as Asia (A), Europe (E), North America (NA), and Central America (CA)^83,84^. The two species of *Tetrops* included in this dataset occur in Europe. However, the genus also includes species from northern Asia. The second analysis focused on *Tetraopes* species. Outgroups were excluded using the drop.tip function from the R package ape^85^. Six biogeographical areas were defined as Arctic (A), Western (W), Alleghany (AL), Mexican Transition Zone (MT), Mesoamerican (M) based on previous studies^86,87^.

### Supplementary Information

Supplementary Information.

## Data Availability

Data and scripts relevant for this project are available at: https://github.com/NayeliGutierrez/2023_MacroevolTetraopes.git. Genome assemblies and UCE custom probes are be available at: https://datadryad.org/stash/landing/show?id=doi%3A10.5061%2Fdryad.gmsbcc2vh.
