# Supplementary material for: Phylogenomics of Tetraopes longhorn beetles unravels their evolutionary history and biogeographic origins
Source: Sci Rep. 2024 Mar 27;14:7285. doi: 10.1038/s41598-024-57827-z (PMC10973435; doi:10.1038/s41598-024-57827-z)

Supporting information

Macroevolutionary patterns behind a classic case of coevolution: Uncovering the evolution of milkweed longhorn beetles (*Tetraopes*, Cerambycidae)

Supplementary Table 1. Specimens included in this study.

Species	Year collected	Preservation method	Country	State	Locality	Collector
**Tetraopini**						
Tetraopes tetrophthalmus	2019	liquid nitrogen	United States	Pennsylvania	Harford County, Maryland	Nayeli Gutiérrez and Josef Vlasak
Tetraopes annulatus	2019	liquid nitrogen	United States	New Mexico	along the road between National Radio Astronomy and Datil	Nayeli Gutiérrez and James Wappes
Tetraopes discoideus	2019	liquid nitrogen	United States	Arizona	Cochise County, Casa de San Pedro	Nayeli Gutiérrez, Gonzalo Martínez, Steve Lingafelter, and Norman Woodley
Tetraopes femoratus	2019	liquid nitrogen	United States	Nebraska	unknown	Thomas Weissling
Tetraopes pilosus	2019	liquid nitrogen	United States	Nebraska	Bessey Rec Complex	Nayeli Gutiérrez and James Wappes
Tetraopes skillmani	2019	liquid nitrogen	United States	Arizona	unknown	Frederick W. Skillman, Jr.
Tetraopes varicornis	2021	ethanol	Mexico	Hidalgo	Tolcayuca Carretera México-Pachuca, Mex 85. After Arco Norte, on the way to México	Cristopher O. Cervantes Meza
Tetraopes cleroides	2021	ethanol	Mexico	Morelos	unknown	Victor Toledo, Nayeli Gutierrez, Gonzalo Martinez
Tetraopes subfaciatus	2021	ethanol	Mexico	Nayarit	unknown	Nayeli Gutierrez, Gonzalo Martinez and Rafael Ceron

Tetraopes quinquemaculatus	2019	ethanol	United States	Nebraska	Howard County Nebraska	Thomas Weissling
Tetraopes melanurus	2021	pinned	United States	New York	Westchester Co. 1.5 mi SE Cross River near end Michigan Road 41.2491N, 73.5922W 19July2021	Robert Naczi
Tetraopes umbonatus	2015	pinned	Mexico	Morelos	Tepoztlan, San Andres de la Cal, Cerro de la Cruz 18.962970N 99.118820W Alt 1573 m Selva Baja Caducifolia	unknown
Tetraopes basalis	2004	pinned	United States	California	Siskyou Co. 1-5 vic Henley 10-VI-2004	Frederick W. Skillman, Jr.
Tetraopes linsleyi	2000	pinned	United States	Arizona	Cochise County, Chiricahua Mts. Horeshoe Canyon 12-VIII-2003	Frederick W. Skillman, Jr.
Tetraopes batesi	1956	pinned	Mexico	Jalisco	W.S. of L. Sayula Aug 3, 1956	Vincent D. Roth
Tetraopes thermophilus	1957, 1947	pinned	United States	Texas	Refugio Co. Oct 18 1957	W. V. Miller
Tetraopes ineditus	2021	ethanol	Mexico	Nayarit	El Guamuchil 13/Ago/2021 20.8151N -105.3893 W	Oscar Perez
Tetraopes sublaevis	unknown	liquid nitrogen	United States	California	unknown	unknown
Tetraopes texanus	1985	pinned	United States	Texas	unknown	unknown
Tetraopes elegans	unknown	pinned	Mexico	Baja California Sur	unknown	unknown
Tetraopes paracomes	unknown	pinned	Mexico	unknown	unknown	unknown
Tetraopes crinitus	1954	pinned	Mexico	Nayarit	unknown	unknown
Tetraopes mandibularis	1986, 1987	pinned	United States	Texas	unknown	unknown
Phaea mankinsi	1960	pinned	Honduras	Comayagua	unknown	unknown
Phaea paralella	2021	ethanol	Mexico	Morelos	Tepoztlan	unknown
Phaea laurieae	2021	ethanol	Mexico	Morelos	Tepoztlan	unknown
Phaea rufiventris	2021	ethanol	Mexico	Morelos	Tepoztlan	unknown
Phaea aff. marthae	2021	ethanol	Mexico	Jalisco	Chamela	unknown

Phaea quadrimaculata	2021	ethanol	Mexico	Morelos	Tepoztlan	unknown
**Tetropini**						
Tetrops praeustus	1972	pinned	Polonia	Piotrków	unknown	unknown
Tetrops starkii	2020	ethanol	Slovakia	Štúrovo	unknown	unknown
**Astathini**						
Bacchisa fortunei	2006	fresh to freezer	Japan	Tokushima	Hachiman	unknown
Tetraophthalmus dimidiatus	2009	pinned	Indonesia	Sumatra	Harau Valley	unknown
Astathes biplagiata	1961, 1951	pinned	India	Karnataka	unknown	unknown
Tropimetopa simulator	2008	pinned	Indonesia	Sumatra	Payakumbuh env., Harau Valley	unknown
Eustathes flava	2012	pinned	The Philippines	Luzon	Nueva Vizcaya, Belance	unknown

Supplementary Table 2. DNA extraction kit and library prep concentrations per species. Species with unknown values were processes by an external laboratory.

	DNA extraction	Library prep	
Species	Kit	Concentration (ng/ul, qubit)	Final lib conc (ng)
**Tetraopini**			
Tetraopes varicornis	DNeasy Blood & Tissue Kit	112.00	2.44
Tetraopes cleroides	DNeasy Blood & Tissue Kit	82.40	3.32
Tetraopes subfaciatus	DNeasy Blood & Tissue Kit	57.20	1.57
Tetraopes quinquemaculatus	DNeasy Blood & Tissue Kit	112.00	4.40
Tetraopes melanurus	Qlamp mini kit	102.00	12.50
Tetraopes umbonatus	Qlamp mini kit	2.34	15.10
Tetraopes basalis	Qlamp mini kit	40.80	1.15
Tetraopes linsleyi	Qlamp mini kit	2.32	27.00
Tetraopes batesi	Qlamp mini kit	0.18	30.20
Tetraopes thermophilus	Qlamp mini kit	1.74	15.80
Tetraopes ineditus	DNeasy Blood & Tissue Kit	57.20	1.57
Tetraopes sublaevis	DNeasy Blood & Tissue Kit	112.00	4.40
Tetraopes texanus	Qlamp mini kit	38.40	2.88
Tetraopes elegans	Qlamp mini kit	0.18	30.20
Tetraopes paracomes	Qlamp mini kit	2.00	6.88
Tetraopes crinitus	Qlamp mini kit	0.15	10.70

Tetraopes mandibularis	Qlamp mini kit	2.32	27.00
Tetraopes tetrophthalmus	MagAttract HMW DNA Kit	NA	NA
Tetraopes annulatus	OmniPrep DNA Extraction kit	NA	NA
Tetraopes discoideus	OmniPrep DNA Extraction kit	NA	NA
Tetraopes femoratus	OmniPrep DNA Extraction kit	NA	NA
Tetraopes pilosus	OmniPrep DNA Extraction kit	NA	NA
Tetraopes skillmani	OmniPrep DNA Extraction kit	NA	NA
Phaea mankinsi	Qlamp mini kit	0.10	2.22
Phaea paralella	Qlamp mini kit	53.20	4.10
Phaea quadrimaculata	Qlamp mini kit	118.00	1.54
Phaea laurieae	Qlamp mini kit	56.20	2.30
Phaea rufiventris	Qlamp mini kit	48.00	6.00
Phaea aff. marthae	DNeasy Blood & Tissue Kit	93.80	3.68
**Tetropini**			
Tetrops praeustus	Qlamp mini kit	1.74	15.80
Tetrops starkii	Qlamp mini kit	93.80	3.68
**Astathini**			
Bacchisa fortunei	DNeasy Blood & Tissue Kit	21.60	2.74
Tetraophthalmus dimidiatus	Qlamp mini kit	49.40	11.90
Astathes biplagiata	Qlamp mini kit	3.40	20.00
Tropimetopa simulator	Qlamp mini kit	61.00	9.68

Eustathes flava	Qlamp mini kit	8.60	14.50

Supplementary Table 3. Genome assembly statistics per species and UCE capture results with identity (50 and min-coverage 67 parameters in PHYLUCE).

	Genome Assembly				UCE Capture		
Species	Total Length (Mbp)	BUSCO	N50	GC (%)	UCEs	Mean Length	95 CI
**Tetraopini**							
Tetraopes tetrophthalmus	771	88.1	30733	32.99	13081	1150.46	0.56
Tetraopes annulatus	648	73.8	5766	32.91	12724	1127.46	0.91
Tetraopes discoideus	615	75.2	5349	32.43	12927	1119.36	0.98
Tetraopes femoratus	715	63.5	3091	33.33	12442	1102.77	1.11
Tetraopes pilosus	682	68.8	4222	32.98	12534	1123.10	0.95
Tetraopes skillmani	677	65.1	3180	32.94	12538	1090.17	1.24
Tetraopes varicornis	534	59.7	2592	31.75	12747	1048.42	1.67
Tetraopes cleroides	312	28.3	1437	30.21	10112	798.85	2.67
Tetraopes subfaciatus	478	33.0	2019	31.42	12224	958.06	2.16
Tetraopes quinquemaculatus	512	59.3	3406	31.79	12740	1054.87	1.69
Tetraopes melanurus	621	68.6	4158	32.77	12739	1110.64	1.10
Tetraopes umbonatus	694	65.6	2531	31.97	12721	1100.13	1.17
Tetraopes basalis	684	79.0	6676	32.82	12877	1136.36	0.81
Tetraopes linsleyi	649	72.9	4400	31.89	12919	1127.59	0.89
Tetraopes batesi	539	22.3	942	31.20	12334	809.19	2.23
Tetraopes thermophilus	526	41.4	1004	32.66	12940	883.15	2.06
Tetraopes ineditus	358	36.1	1688	30.39	11817	885.29	2.35
Tetraopes sublaevis	246	4.2	1015	31.02	6910	652.84	3.06
Tetraopes texanus	108	29.3	1407	48.33	7735	472.09	1.94

Tetraopes elegans	397	13.0	841	29.86	6892	621.55	2.86
Tetraopes paracomes	36	3.3	3080	54.88	512	356.10	3.17
Tetraopes crinitus	261	4.4	714	41.32	5822	456.79	1.93
Tetraopes mandibularis	230	12.5	681	38.58	11039	576.91	2.00
Phaea mankinsi	189	7.9	1529	51.30	1059	348.27	2.16
Phaea paralella	362	12.7	1078	31.72	10036	731.43	2.60
Phaea quadrimaculata	503	33.0	1617	31.61	11825	910.14	2.35
Phaea laurieae	362	71.3	4334	31.06	12784	1065.14	1.59
Phaea rufiventris	397	46.3	1845	31.48	12369	944.34	2.17
Phaea aff. marthae	349	84.0	7276	31.70	12912	1111.21	1.22
**Tetropini**							
Tetrops praeustus	140	7.3	669	37.41	10634	534.25	1.86
Tetrops starkii	418	76.4	4504	33.31	12854	1098.00	1.35
**Astathini**							
Bacchisa fortunei	577	36.6	1774	30.88	11464	912.87	2.39
Tetraophthalmus dimidiatus	591	34.4	820	33.44	12649	779.83	2.09
Astathes biplagiata	604	38.4	1150	30.66	12600	879.61	2.18
Tropimetopa simulator	319	25.6	801	33.14	12473	728.43	2.07
Eustathes flava	434	45.2	1900	46.40	11519	660.02	2.14

Supplementary List 1. List of the fifty morphological characters included in the phylogenetic analysis.

1. Body, total length. Coded as continuous.
2. Integument of the body (dorsally) 0: Bicolored 1: Red
3. Integument of the pronotum (not considering the black maculae next to the umbone and the umbone): 0: Bicolored 1: Red 2: Black 3: Orange
4. Integument of the elytra (not considering the black maculae next to the umbone): 0: Bicolored 1: Red 2: Black
5. Apical third of the labrum in lateral view: 0: Convex 1: Oblique
6. Mandibles: 0: Sexually dimorphic 1: Sexually monomorphic
7. Last segment of maxillary palpi: 0: Slender, tapering 1: Inflated
8. Lower eyes lobes: 0: Longer or subequal to genae 1: Shorter than genae
9. Upper interocular space in females: 0: Narrower than the length of the scape 1: Wider than the length of the scape
10. Antennae: 0: Not annulate 1: Annulate
11. Antennae: 0: With dorsal and ventral pubescence of same color 1: With dorsal and ventral pubescence of different color
12. Antennae: 0: With ventral and dorsal pubescence of same length 1: With ventral pubescence shorter than dorsal 2: With dorsal pubescence shorter than ventral

13. Antennae in males: 0: Shorter than body 1: Longer than body

14. Scape: 0: Shorter than third segment 1: Same length or longer than third segment

15. Apex of scape: 0: Uniform 1: Asperate

16. Apex of the last antennal segment: 0: Acute 1: Obtuse

17. Apical third of the pronotum: 0: Not impressed 1: Impressed

18. Sides of the pronotum: 0: Not lobed 1: Lobed 2: With acute spines

19. Sides of the pronotum: 0: Without black spots 1: With a black spots

20. Pronotum: 0: With umbone abruptly elevated and distinctly delimited 1: With umbone not abruptly elevated and not distinctly delimited 2: Without umbone

21. Pronotum: 0: With umbone base extending to reach anterior depression 1: With umbone base surpassing anterior depression 2: Without umbone

22. Pronotum 0: Umbone with pubescence and setae intermingled 1: Umbone with only setae; 2: Umbone with only pubescence 3: Without umbone

23. Pronotum 0: Umbone with no setae 1: Umbone scarce setae 2: Umbone dense setae 3: Without umbone

24. Pronotum: 0: Umbone with scarse punctuation in the center 1: Umbone with dense punctuation in the center 2: Without umbone

25. Pronotum: 0: With scarse pubescence in the center 1: With dense pubescence in the center 2: Without pubescence

26. Pronotum: 0: Without dark spots at sides of umbone 1: With dark spots at sides of umbone 2: Without umbone

27. Elytra: 0: More than twice as long as wide 1: Less than twice as long as wide

28. Sides of the elytra: 0: Straight 1: Impressed

29. Elytral humeri: 0: Without maculae 1: With maculae

30. Basal punctures of elytra: 0: Coarse 1: Fine

31. Basal punctures of elytra: 0: Dense 1: Sparse

32. Elytral punctures: 0: Linearly arranged 1: Irregularly arranged

33. Elytra: 0: Covered with pubescence and erect setae 1: Covered only with appressed pubescence 2: Covered only with erect setae

34. Pubescence of elytra: 0: Obscuring integument 1: Not obscuring integument 2: Without pubescence

35. Elytral disk (at base of apical third) 0: Without black spots 1: With glabrous black spots

36. Elytra 0: Without a median lateral macula 1: With a median lateral macula

37. Middle third of the elytra: 0: Without spots in the discal area 1: With black pubescence circular spots

38. Postmedian elytra: 0: Without black spots 1: With black spots of pubescence 2: With black lines of pubescence

39. Elytra: 0: Without chevrons 1: With two discontinuous chevrons 2: With two continuous chevrons

40. Elytral disk: 0: Without dark chevrons 1: With dark chevrons covered with bluish pubescence 2: With dark chevrons covered with whitish pubescence

41. Elytral disk: 0: Without chevrons 1: With chevrons extending apically to the elytral declivity 2: With chevrons not extending apically to the elytral declivity

42. Elytral disk: 0: Without chevrons 1: With chevrons extending laterally to the elytral declivity 2: With chevrons not extending laterally to the elytral declivity

43. Elytral disk: 0: Without chevrons 1: With apices of chevrons extending to the scutellum 2: With apices of chevrons not extending to the scutellum

44. Femora (at least pro- and mesofemora): 0: yellowish to brown 1: piceous to black 2: red 3: blue and black

45. Apices of posterior femora in females: 0: Not surpassing the posterior margin of the second abdominal segment 1: Surpassing the margin of the second abdominal segment.

46. First tarsal segment: 0: Shorter than second and third segments together 1: Equal to or longer than second and third segments together

47. Claws: 0: Bifid 1: Appendiculate

48. Fifth abdominal sternite: 0: Longer than 3 + 4 1: Shorter or subequal in length to 3 + 4

49. Eyes: 0: Completely separated 1: Not completely separated

50. Front of head 0: vertically inclined to longitudinal axis of body 1: obliquely or subvertically inclined to longitudinal axis of body

Supplementary List 2. Matrix with fifty-one morphological characters and 12 species formatted for analysis in TNT.

```
nstates 32;
xread
51 12

&[cont]
Tetraopes_annulatus 9.45-12.6
Tetraopes_discoideus 7.8-10.2
Tetraopes_femoratus 10.5-15.3
Tetraopes_pilosus 13.5-16.65
Tetraopes_skillmani 7.65-8.1
Tetraopes_tetrophthalmus 13.35
Phaea_saperda 7.8-9.9
Tetrops_rottensis 3.15
Tetrops_praeustus 3.0-6.0
Anoplophora_glabripennis 20.0-35.0
Tetraopes_batesi 7.8-9.6
Tetraopes_umbonatus 8.25-12.6

&[num]
Tetraopes_annulatus 111010110100010011010010011100011110010000000211000
Tetraopes_discoideus 000[12]10010112110110001020111100010110011212?1110100
Tetraopes_femoratus 111110010102010011000020011111[01]01[01][01]110100000[12]211000
Tetraopes_pilosus 111110110102010011010210111111111111001000000210000
Tetraopes_skillmani 000210100002110000001021111100010000001?11??1110100
Tetraopes_tetrophthalmus 111110110002010111110020011111010011010000000110000
Phaea_saperda     030001000000101111000130000000000000000000000000100
Tetrops_rottensis ???????????????1?1????????0??????????????????????0
Tetrops_praeustus 020201101001000010022332220000012200000000000?00100
Anoplophora_glabripennis 0222???0?1??10?1?20222322201011110000000000003001?10
Tetraopes_batesi 01011?110102?1010000102101110001010001212121110100
Tetraopes_umbonatus 010110110012110000001021111100010000011122221110100

;
proc/;
```

Supplementary Figure 1. Single most parsimonious tree based on morphological characters. Image sources: [19, 87].

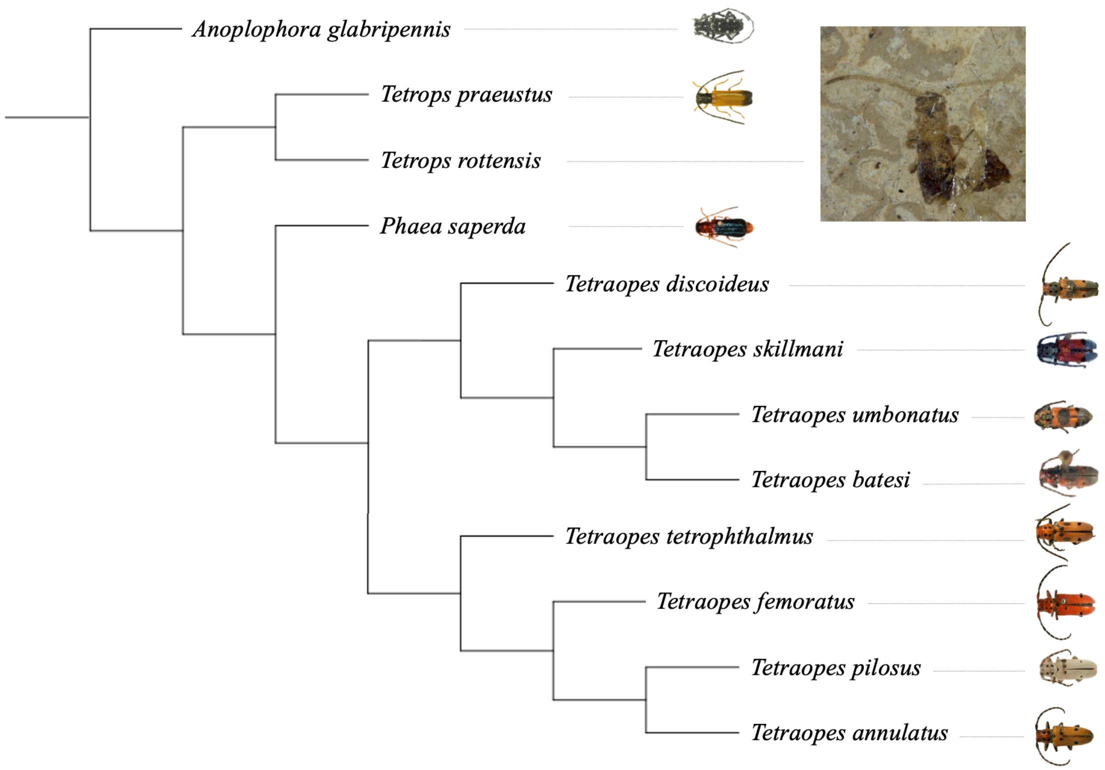

Supplement: Supplementary file 1 — Supplementary Information. [file 41598_2024_57827_MOESM1_ESM.pdf]
